# Supplementary material for: Identification of photoperiod-induced specific miRNAs in the adrenal glands of Sunite sheep (Ovis aries)
Source: Front Vet Sci. 2022 Jul 22;9:888207. doi: 10.3389/fvets.2022.888207 (PMC9354845; doi:10.3389/fvets.2022.888207)
Supplement: Supplementary file 1 [file Data_Sheet_1.ZIP › Supplementary file/miRDeep2 file.zip!/miRDeep2 file/Know_Structure/LP42A2/oar-mir-200a.pdf]

miRBase precursor	: oar-mir-200a
Total read count	: 433
oar-miR-200a read count	: 422
remaining reads	: 11

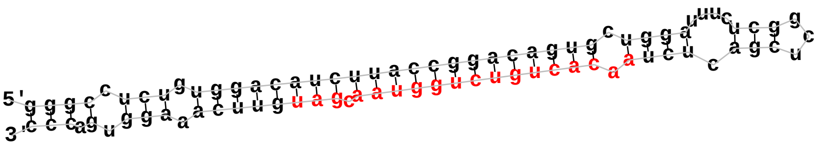

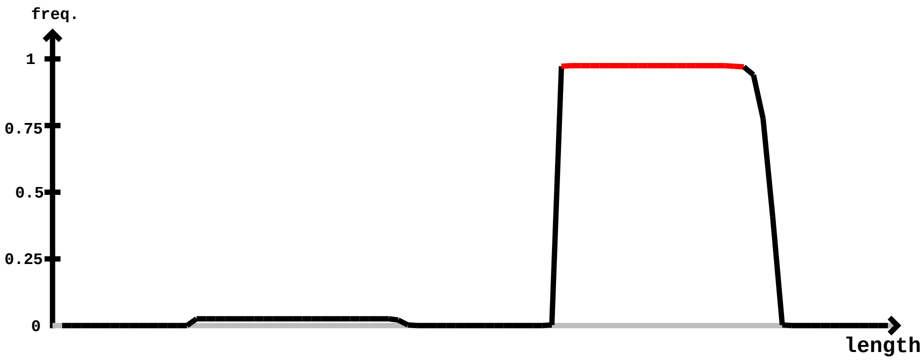

oar-miR-200a

```
5'- gggccucuguggacaucuuaccggacagugcuggauuucucggcucgacucuaacacugucugguaacgauguucaaaggugaccc   -3'    exp
    ((((.(((.(((((((((((((((((((((.((((....(((...))).)))).))))))))))))).)))))))).))).).)))    reads       mm          sample
    .............caucuuaccggacagugcugg....................................................    2           0           seq
    .............caucuuaccggacagugcugga...................................................    8           0           seq
    .............caucuuaccggacagugcuggau..................................................    1           0           seq
    ..................................................cuaacacugucugguaacgauguu............    1           0           seq
    ...................................................uaacacugucugguaaAg.................    1           1           seq
    ...................................................uaacacugucugguaacga................    1           0           seq
    ...................................................uaacacugucugguaaAgau...............    5           1           seq
    ...................................................uaacacugucugguaacgau...............    8           0           seq
    ...................................................uaacacugucugguaacgaug..............    51          0           seq
    ...................................................uaacacugucugguaaAgaug..............    20          1           seq
    ...................................................uaacacugucugguaacgaugu.............    158         0           seq
    ...................................................uaacacugucugguaacgauguu............    175         0           seq
    ...................................................uaacacugucugguaacgauguuc...........    1           0           seq
    ....................................................aacacugucugguaacgaugu.............    1           0           seq
```
